# After the epidemic: Zika virus projections for Latin America and the Caribbean

**DOI:** 10.1371/journal.pntd.0006007

**Published:** 2017-11-01

**Authors:** Felipe J. Colón-González, Carlos A. Peres, Christine Steiner São Bernardo, Paul R. Hunter, Iain R. Lake

**Affiliations:** 1 School of Environmental Sciences, University of East Anglia, Norwich, Norfolk, United Kingdom; 2 Universidade do Estado de Mato Grosso, Rua São Pedro s/n, Cavalhada, Cáceres, Mato Grosso, Brazil; 3 Norwich Medical School, University of East Anglia, Norwich, Norfolk, United Kingdom; Federal University of Agriculture, NIGERIA

## Abstract

**Background:**

Zika is one of the most challenging emergent vector-borne diseases, yet its future public health impact remains unclear. Zika was of little public health concern until recent reports of its association with congenital syndromes. By 3 August 2017 ∼217,000 Zika cases and ∼3,400 cases of associated congenital syndrome were reported in Latin America and the Caribbean. Some modelling exercises suggest that Zika virus infection could become endemic in agreement with recent declarations from the The World Health Organisation.

**Methodology/Principal findings:**

We produced high-resolution spatially-explicit projections of Zika cases, associated congenital syndromes and monetary costs for Latin America and the Caribbean now that the epidemic phase of the disease appears to be over. In contrast to previous studies which have adopted a modelling approach to map Zika potential, we project case numbers using a statistical approach based upon reported dengue case data as a Zika surrogate. Our results indicate that ∼12.3 (0.7–162.3) million Zika cases could be expected across Latin America and the Caribbean every year, leading to ∼64.4 (0.2–5159.3) thousand cases of Guillain-Barré syndrome and ∼4.7 (0.0–116.3) thousand cases of microcephaly. The economic burden of these neurological sequelae are estimated to be USD ∼2.3 (USD 0–159.3) billion per annum.

**Conclusions/Significance:**

Zika is likely to have significant public health consequences across Latin America and the Caribbean in years to come. Our projections inform regional and federal health authorities, offering an opportunity to adapt to this public health challenge.

## Author summary

In February 2016 the World Health Organisation (WHO) declared Zika virus infection in the Americas as a Public Health Emergency of International Concern (PHEIC). By November 2016, Zika was declared a long-term public health challenge. This change of status implies that Zika is likely to become an endemic problem in the region. Due to the PHEIC status of Zika, most current research has rightly focused on the epidemic stage of the disease; however, it is timely and critical to consider the public health consequences after such epidemic phase. We used one of the largest and most spatially diverse panels of epidemiological surveillance data comprising 12 years of dengue case observations from Brazil and Mexico, and covering an area of over ten million km^2^. State-of-the-art statistical models, and high-resolution (0.5 × 0.5 degrees) climate and demographic data were used to produce spatially-explicit projections of Zika infection for Latin America and the Caribbean. Model projections were then used to estimate the number of cases with neurological sequelae and their economic cost. Our findings indicate that the potential health and economic burden of Zika could be considerably large for the region should it become endemic. The estimated burden of Zika under an endemic state highlights the need for health authorities in the countries at risk to promote preventive and control measures.

## Introduction

Zika virus (ZIKV) is a vector-borne disease that is transmitted among humans through the bite of infectious *Aedes* mosquitoes. ZIKV is a member of the Flaviviridae family, and genus *Flavivirus*. The symptoms of ZIKV infection are usually mild and similar to those of other arboviral infections such as dengue including fever, macopapular rash, conjunctivitis, myalgia, and headache [1]. In most infected people the disease is benign. However, in some cases ZIKV infection may result in serious complications such as Guillain–Barré syndrome, microcephaly and maculopathy [2–4]. To the date of this study, there are no vaccines or antiviral therapy readily available for ZIKV infection [5]. However, this could be feasible in the future [6].

In April 2015, a ZIKV outbreak was reported in Brazil, and subsequently in several Latin American and Caribbean countries. By 3 August 2017 ∼217,000 confirmed ZIKV cases, and ∼3,400 cases of associated congenital syndrome had been reported to the Pan-American Health Organization [7]. Current research on ZIKV activity has rightly focused upon the disease epidemic stage [8] and its consequences. The next big question is whether Zika will become endemic in Latin America and the Caribbean (LATAM), and what are the potential health and economic burdens.

Although it is impossible to ascertain whether ZIKV will become endemic in LATAM, a recent study based on a numerical epidemic model predicts that the virus will eventually become endemic [9]. The lack of vaccines for ZIKV [5], the environmental suitability of the region [10], and the endemic status of other arboviruses that share the same vector (e.g. dengue fever) also suggest that such an endemic state is plausible.

One key aspect for the control of mosquito-borne diseases is vector control. Past experience indicates that aggressive control of *Aedes* mosquitoes using traditional insecticide-based measures is effective only if implemented in a comprehensive and sustained manner [11]. This may be difficult due to public resistance, lack of expertise, and finance [11, 12]. A recent meta-review on the effectiveness of *Aedes* control strategies has found that this type of vector control does not seem to be associated with long-term reductions of mosquito populations [13]. Physical control measures against the vector such as house screens, and the environmental modification or sanitation of larval sites may also be effective [14]; however, these measures may be unavailable to poor residents in crowded urban areas where the impacts of ZIKV are greatest [5].

Recent studies have mapped the potential global scale range of ZIKV based on combinations of environmental, vector abundance, and socioeconomic factors [10, 15]. One limitation of the method used by these studies is that it maps the environmental suitability which does not necessarily imply that the disease will occur in that area [10]. This issue is critical because experience from similar diseases indicates that such modelling approaches tend to overestimate the geographical areas where disease could occur as they cannot take into account the complex local factors that determine whether potential risk actually translates into disease [16]. An alternative approach is a statistical analysis based on spatially-explicit monthly reports of confirmed ZIKV cases; a difficult task due to the limited time period for which reliable human spatially-explicit case reports are available for LATAM [17–19].

We overcome these limitations by using human dengue case data across LATAM as a surrogate for ZIKV. The advantage of this approach is that it is based upon knowledge on where disease transmission from mosquito to humans occurs in reality. One challenge, however, is that reported disease counts are a fraction of the true incidence (it has been estimated that for each official dengue report ∼10–27 cases go unreported [20]). We argue that this approach is valid because the dengue virus shows remarkable similarities to ZIKV. For example, both viruses have single positive stranded RNA genome encoding three structural proteins (C, prM/M and E), and seven non-structural proteins (NS1, NS2A, NS2B, NS3, NS4A, NS4B, and NS5); are vectored by *Aedes* mosquitoes; and seem to have similar infectious and viral replication mechanisms [21].

Moreover, phylogenetic analyses have shown that ZIKV is closer to dengue virus than to any other flavivirus [22]. As a consequence of such similarities, there is cross-reaction of antibodies to dengue with ZIKV [23]. Not surprisingly, previous ZIKV modelling studies are largely based upon dengue parameters [8, 24]. We acknowledge, however, that whilst there is only one ZIKV serotype, there are four different dengue serotypes which do not confer protective immunity against all serotypes [6]. Thus, while Zika could infect an individual only once, dengue could cause disease repeatedly which poses a key difference in the ecology and epidemiology of these two diseases.

The aims of our work are three-fold. Focusing upon the post-epidemic period of ZIKV, we first examine the likely incidence of ZIKV in childbearing women across LATAM and the potential number of microcephaly and Guillain-Barré syndrome (GBS) cases. Second, we identify areas where ZIKV transmission may be sporadic and hence remains epidemic re-emerging every few years. Finally, we quantify how case numbers are likely to fluctuate in affected areas due to seasonal and meteorological effects such as an El Niño (ENSO) event.

## Materials and methods

### Epidemiological surveillance data

Monthly counts of laboratory confirmed dengue cases were obtained from the Mexican [25], and Brazilian [26] Ministries of Health for the period January 2001 to December 2012 (144 months). Together, these two countries cover a latitudinal range between 30°N and 30°S, and account for over 60% of the reported dengue cases and ∼53% of the LATAM population. Our dataset consists of nearly 4 million dengue reports (Brazil 88%, Mexico 12%). The Mexican dataset was obtained at the State level (n = 32, mean population = 3.2 million people), whilst the Brazilian dataset was obtained at the municipal county level (n = 5,566, mean population = 0.47 million people). Missing counts were imputed for municipalities with less than 20% missing entries using a singular value decomposition-based method [27], included in the *bcv* package [28] for R [29]. Areas with over 20% missing counts (n = 4,177) were removed from the dataset. Brazilian municipal counties are considerably smaller in area and population than the Mexican States. Such small areas were typically characterized by low counts of cases. We aggregated the Brazilian municipal counties into larger geographical units by dividing the centroid coordinates into 286 latitude-longitude intervals, and merging all counties with centroid coordinates within each latitude-longitude bin together. The merged areas (n = 286, mean population = 0.45 million people) were used for analysis.

Whilst the presence of four dengue serotypes is an important difference with ZIKV that we acknowledge, it would be impossible to disentangle the dengue epidemiological surveillance data to obtain four different time series, one for each serotype. The ratio of dengue to ZIKV cases is hard to estimate due to the limited period for which ZIKV data are available. Given that the transmission dynamics of ZIKV and dengue are similar when observed in the same setting [30], for simplicity, we initially assumed that each confirmed dengue report is equivalent to a ZIKV case (1:1 ratio). To account for uncertainties in this assumption, we also considered scenarios where the ZIKV to dengue ratio varied between 0.1:1 and 10:1.

### Meteorological data

High-resolution gridded datasets of monthly global mean temperature, total precipitation, and potential evapotranspiration (PET) data [31] were obtained from the CRU TS3.24 Climatic Research Unit climate archives at a 0.5 × 0.5 degree resolution for land cells only, and for the period January 1991 to December 2015. Moving averages were computed for the current and previous two months to account for the delayed effects of temperature, precipitation, and PET on incidence [32]. Mean temperature, mean PET, and total precipitation estimates for each administrative unit in the study were calculated using the *extract* method included in the R [29] *raster* package [33].

### Demographic data

Global gridded total population count estimates were retrieved at a 2.5 arc minutes resolution from the Gridded Population of the World project [34] at five year intervals for the period 2000–2010. For consistency with the meteorological data, demographic data were aggregated at a 0.5 × 0.5 degree resolution using the Climate Data Operators software [35]. Total population estimates were scaled to agree with the United Nations World Population Prospects yearly population estimates [36]. Monthly estimates for each grid-box were derived using linear interpolation [32, 37]. The estimated population for each geographical unit included in the study was then calculated using the *extract* method included in the R [29] *raster* package [33]. Crude birth rates per country were also retrieved from the United Nations World Population Prospects [36].

### Model specification

The expected number of Zika virus infections *E*(*Y*_*it*_) for area *i* = 1, …, *I* at time *t* = 1, …, *T* was modelled using a generalized additive mixed model (GAMM) approach. To account for possible over-dispersion in the data, we fitted Negative Binomial and quasi-maximum likelihood Poisson models. We selected the model specification with the lowest mean absolute error (MAE). The general algebraic definition of both the Negative Binomial and quasi-maximum likelihood Poisson models is given by:

$$\begin{array}{c} log\left(\mu_{it}\right)=\eta_{it} \end{array}$$ (1)

$$\begin{array}{c} \eta_{it}=\alpha +Log\left(\xi_{it}\right)+t^{\prime }+s^{\prime }+\sum_{p=1}^{P}f\left(x_{it}\right)+d_{i} \end{array}$$ (2)

where *η*_*it*_ is a logarithmic link function of the expectation *E*(*Y*_*it*_ ≡ *μ*_*it*_), with *Y*_*it*_ as the time series of monthly dengue reports. The term *α* corresponds to the intercept; *Log*(*ξ*_*it*_) denotes the logarithm of the population at risk for area *i* and time *t* included as an offset to adjust the epidemiological data by population. Here, *t*′ is a cubic regression spline function of the time variable with 1 degree of freedom (*df*) for every *M* years of data to control for possible long-term trends. Seasonal trends are modelled using Fourier terms (*s*′) with *N* sine/cosine pairs. Long-term and seasonal trends in all variables in the model are controlled for because they may be related to factors other than climate [38] such as changes in reporting or coverage, holidays or seasonal water storage. The term *f*(*x*_*it*_) corresponds to smoothed relationships between the climatic predictors and the crude incidence rate defined by the cubic regression splines. Area-specific random effects (*d*_*i*_) were included to account for the effects of unknown or unobserved variables in the model such as diagnostic performance variability, immunity, and intervention measures.

### Selection of climatic predictors

A time series cross-validation (TSCV) algorithm [39] was implemented to select the set of climate predictors producing the lowest prediction error. TSCV was preferred over k-fold or leave-one-out cross-validation algorithms because epidemiological surveillance time series are typically serially correlated [40] violating the assumptions that data are independent and identically distributed. Models were fitted using all climatic predictors (i.e. mean monthly temperature, mean monthly PET and total monthly precipitation) in isolation, as well as in all possible combinations. Therefore, we successively fitted all possible models containing one climatic predictor at a time, then two predictors at a time, and so on, until all predictors were included altogether in a single model. We measured the accuracy of each model calculating their MAE. The MAE was selected as the measure for model accuracy because it is a natural and unambiguous measure of average error magnitude [41].

TSCV was implemented dividing the dataset into a training and a test sets. The initial training set comprised 90% of the total number of months (*n* = 144). Each time step (*k*), a further month of data was added to the training set. Thus, at time step *k* = 1, the training set comprised observations for month *t* = 1, …, 130; at *k* = 2 it comprised observations for *t* = 2, …, 131, and so on. The test set comprised the first observation for each geographical area immediately after the last observation in the training set. Consequently, at time step *k* = 1, the test set contained all area-specific observations for *t* = 131; at *k* = 2, it contained all observations for *t* = 132, and so on until the test set contained the observations for month *t* = *n*; where *n* is the total number of months in the dataset. The MAE was calculated at each time step *k* = 1, …, *K*, and for each subset of climatic predictors *h* = 1, …, *H* as in the following matrix:

$$\begin{array}{c} MAE_{k,h}=[\begin{array}{cccc} MAE_{1,1} & MAE_{1,2} & \cdots & MAE_{1,H}\\ MAE_{2,1} & MAE_{2,2} & \cdots & MAE_{2,H}\\ \vdots & \vdots & \ddots & \vdots \\ MAE_{K,1} & MAE_{K,2} & \cdots & MAE_{K,H} \end{array}] \end{array}$$

The MAE for each modelled subset (henceforth MAE_*k*,*h*_) was calculated by averaging the subset-specific values (*h*) across all time steps (*k*). With this process, we aimed to identify the most accurate model or group of models.

### Specification of the long-term and seasonal trends

TSCV was used to identify the specification of long-term and seasonal trends with the lowest MAE_*k*,*h*_. Specifically, we modified the number of *df* per year (ranging from 1 *df* for every two years of data to 1 *df* for every four years) for the cubic spline function of time, as well as the number of sine/cosine pairs for the Fourier terms (ranging from three to six). All possible combinations of long-term and seasonal trends were explored.

### Model predictions for Latin America

Cross-validated model outputs were used to predict the total number of ZIKV infections for an average month, a typical ENSO month, a strong El Niño (based on the 1997–1998 and 2015–2016 events) [42], and a typical non-El-Niño month across LATAM under the assumption of a 0.1:1, 1:1 and 10:1 ZIKV to dengue ratios. To account for uncertainties in the under-reporting of the health data, we multiplied the predicted number of cases for a given geographical area by a factor of 10, 18.5 or 27 [20]. Model predictions were computed using mean monthly gridded climatic and population data at a 0.5 × 0.5 degree resolution for each of the aforementioned periods. ENSO events are defined here as periods where the 3-month running mean of the Oceanic Niño Index is greater than 0.5°C. The length of an ENSO event was the length indicated by the USA National Weather Service, Climate Prediction Center [43] plus three months to account for potential delayed effects on the local climate. Country-wide totals were retrieved using standard routines within the *raster* [33] R package.

### Estimating the risk of neurological sequelae and their economic impact

Model estimates of mean monthly cases were downscaled by the proportion of cases occurring in childbearing women (i.e. 15–44 years of age) based on the proportion of cases per gender and age reported to the Mexican Ministry of Health over the period 2010–2012 [25]. The number of cases in childbearing women was then used to estimate the potential number of ZIKV-affected pregnancies by multiplying them by the corresponding country-specific crude birth rates [36]. The risk of microcephaly due to ZIKV infection during the first trimester of pregnancy was calculated using the 0%, 50% and 100% percentiles of the distribution of the range of values estimated for the risk of microcephaly due to infection in women aged 15–44 (i.e. 0.88–14.4) [44] to account for uncertainties on our estimates. Similarly, the potential number of GBS cases in both males and females was estimated using the 0%, 50% and 100% percentiles of the distribution of risk estimates of GBS per 1000 ZIKV infections based on previous research conducted in French Polynesia and LATAM [3, 45].

The economic impact of the estimated number of cases with neurological sequelae was estimated based on the direct medical cost of each microcephaly and GBS case [46]. Thus, the estimated mean annual number of microcephaly (*X*) and GBS cases (*Y*) for each administrative unit (*i*) was multiplied by the estimated medical cost per case based on previous research [46]. The direct medical cost of each microcephaly case (*δ*) was assumed to be USD 91,102 whilst that of each GBS case (*γ*) was assumed in USD 28,818 [46]. The total economic impact of the neurological sequelae was estimated as follows:

$$\begin{array}{c} Microcephaly_{i}=X_{i}\;\times \;\delta \end{array}$$ (3)

$$\begin{array}{c} GBS_{i}=Y_{i}\;\times \;\gamma \end{array}$$ (4)

$$\begin{array}{c} Total\;Cost_{i}=Microcephaly_{i}\;+\;GBS_{i} \end{array}$$ (5)

### Identification of epidemic-prone regions

Epidemic-prone areas were defined as areas where the month-to-month relative standard deviation (RSD) of the model estimates is greater than the mean for a given grid box. The RSD is defined here as the ratio of the standard deviation (*σ*) to the mean (*μ*). We defined epidemic areas as those where the RSD of the estimated number of cases was larger than one, and highly epidemic areas where this ratio was greater than 1.5 [47].

$$\begin{array}{c} RSD=\sigma /\mu \end{array}$$ (6)

## Results/Discussion

Previous studies have used combinations of environmental, vector abundance, and socioeconomic factors to map the environmental suitability for ZIKV [10, 15]. However, the fact that a region is environmentally suitable for transmission does not necessarily imply autochthonous transmission will necessarily occur in that area [10]. Recent research suggests that such modelling approaches overestimate the geographical areas where disease is likely to occur [19]. The lack of long-term spatially-explicit monthly reports of confirmed ZIKV cases for LATAM [17–19] poses serious difficulties for the development of alternative approaches based on ZIKV epidemiological surveillance. To overcome these limitations, we used one of the largest panels of epidemiological surveillance dengue case time series for LATAM as a surrogate for ZIKV. Compared to models aimed to predict the environmental suitability for ZIKV, our approach has the advantage of being based upon knowledge of where disease transmission really occurs.

### Model output

We fitted 23 different model specifications to test all possible combinations of climatic predictors long-term and seasonal trends whilst assuming a ZIKV-dengue ratio of 1:1. The TSCV algorithm applied to the dengue-derived ZIKV data (henceforth ZIKV data) favoured a Negative Binomial GAMM with a *MAE*_*k*,*h*_ of 105 cases per month that included temperature lagged zero to two months (*T*_0:2_), and PET lagged zero to two months (*PET*_0:2_) as climatic covariates. Precipitation lagged zero to two months was not included in the final model. The incorporation of an interaction term between *T*_0:2_ and *PET*_0:2_ did not increase the predictive ability of the model, and so it was not included in the final model. After performing a sensitivity analysis testing different specifications for the df of long-term and seasonal trends, the long-term trends in the final model were specified with a cubic regression spline with three df, and the seasonality was specified with a Fourier term with three sine and cosine functions of time. The final model explained 79.5% of the deviance in the health data. The structure of the final model was then used to compute estimates based on both 0.1:1 and 10:1 ZIKV-dengue ratios.

S1 Fig compares the observed and predicted temporal trends in the number of cases for each country. We noted that the final model’s predictions (and their corresponding error estimates) capture quite closely the temporal variations observed in the observed data with some underestimations in both countries related to major outbreaks that could be related to location-specific non-climatic factors (e.g. human behaviour and interventions) not explicitly accounted for in the model [16].

GAMMs are essentially a nonparametric method; therefore, it is difficult to express their results using mathematical equations. Instead, the GAMM-estimated smoothed relationships between ZIKV incidence, *T*_0:2_ and *PET*_0:2_ are presented in S2 Fig. The solid lines in the figure represent the estimated functional form of the relationship between ZIKV incidence and each predictor. S2A Fig shows an almost null response of ZIKV to *T*_0:2_ below 20°C, with rapid increases in ZIKV cases as *T*_0:2_ surpasses this threshold. The estimated effect is consistent with the biology of both the vector and ZIKV because rising temperatures shorten the development time and gonotrophic cycle of the vector, and increase its biting rate; also, they reduce the time required for viral development inside the vector all of which results in an increased risk of transmission [42, 48]. S2B Fig indicates that there is a log-negative relationship between ZIKV incidence and *PET*_0:2_ with the risk of infection drastically decreasing between one and three mm per month and remaining low after that threshold. We were unable to identify studies investigating the effects of PET on ZIKV or *Aedes* mosquitoes. However, previous research using anopheline data [49] has shown similar relationships between PET and vector abundance with low levels being more conducive of vectorial activity than high PET levels, and so increasing the risk of disease transmission. High temperatures and low humidity levels have been found to reduce the oviposition rate and life span of *Aedes* mosquitoes [50].

### Model predictions for Latin America and the Caribbean

We then used the model output to predict the mean monthly number of cases across LATAM for a typical year at a 0.5 × 0.5 degree resolution. A sensitivity analysis was performed to compute predictions under the assumption of a 0.1:1, 1:1, and 10:1 ZIKV to dengue ratio to explore the uncertainties in our assumptions of the relationship between the estimated number of ZIKV and dengue virus infections. Based on such sensitivity analysis, we estimate that should ZIKV become endemic ∼12 million (range: 713 thousand to 162 million) ZIKV cases could occur across LATAM every year (Table 1). About 4 million of those cases (range: 99 thousand to 85 million) are expected to occur in childbearing women (15–44 years of age) annually. The country-level estimates suggest that Brazil will experience the largest disease burden (60%); more than six times the estimated burden for Mexico (7%) or any other LATAM country (Table 1). Other countries such as Colombia, Mexico, Venezuela, Cuba and Peru are also expected to experience large numbers of ZIKV infection.

**Table 1:** Country-level projections of Zika virus (ZIKV) infection under the assumption of endemicity for the total population and women on childbearing age, and estimated health and economic burden of Guillain-Barré syndrome, and microcephaly. Values in brackets represent the mean estimates under the assumption of a 0.1:1 and 10:1 ZIKV-dengue ratios.

Country	Cases (Thousand)	Cases women 15-44 (Thousand)	Guillain-Barré (Individuals)	Affected pregnancies (Individuals)	Microcephaly (Individuals)	Economic impact (Million USD)
Brazil	7344 (426–95899)	2300 (59–50096)	38335 (102–3047671)	34499 (2001–450544)	2636 (18–64878)	1345 (5–93738)
Colombia	1153 (67–15209)	361 (9–7914)	6020 (16–483352)	5655 (327–74642)	432 (3–10748)	213 (1–14908)
Mexico	1094 (62–14785)	343 (9–7745)	5708 (15–469855)	6207 (355–83840)	474 (3–12073)	208 (1–14640)
Venezuela	841 (48–11453)	263 (7–6014)	4391 (12–363991)	4952 (284–67556)	378 (2–9728)	161 (1–11376)
Cuba	284 (17–3708)	89 (2–1927)	1484 (4–117833)	935 (55–12193)	71 (0–1756)	49 (0–3556)
Peru	212 (12–2722)	66 (2–1420)	1104 (3–86512)	1228 (72–15837)	94 (1–2281)	40 (0–2701)
Dominican Republic	176 (10–2305)	55 (1–1190)	918 (2–73253)	1179 (69–15449)	90 (1–2225)	35 (0–2314)
Guatemala	168 (9–2291)	53 (1–1189)	878 (2–72803)	1013 (57–13870)	77 (1–1997)	32 (0–2280)
Haiti	108 (6–1403)	34 (1–724)	562 (2–44582)	824 (49–10726)	63 (0–1545)	22 (0–1425)
Ecuador	105 (6–1409)	33 (1–725)	546 (1–44779)	644 (36–8692)	49 (0–1252)	20 (0–1404)
Panama	102 (6–1311)	32 (1–679)	531 (1–41653)	612 (37–7862)	47 (0–1132)	20 (0–1304)
Bolivia	96 (6–1280)	30 (1–666)	503 (1–40691)	660 (38–8762)	50 (0–1262)	19 (0–1288)
Argentina	87 (5–1201)	27 (1–634)	453 (1–38161)	332 (18–4563)	25 (0–657)	15 (0–1160)
Costa Rica	81 (5–1076)	26 (1–555)	425 (1–34198)	281 (16–3724)	21 (0–536)	14 (0–1034)
Puerto Rico	72 (4–925)	23 (1–480)	376 (1–29397)	273 (16–3506)	21 (0–505)	13 (0–893)
El Salvador	69 (4–909)	22 (1–467)	361 (1–28883)	184 (11–2416)	14 (0–348)	12 (0–864)
Nicaragua	68 (4–912)	21 (1–473)	355 (1–28973)	392 (22–5264)	30 (0–758)	13 (0–904)
Paraguay	61 (3–843)	19 (0–436)	317 (1–26790)	268 (14–3737)	20 (0–538)	11 (0–821)
Jamaica	56 (3–709)	17 (0–366)	291 (1–22544)	308 (18–3910)	24 (0–563)	11 (0–701)
Guyana	52 (3–668)	16 (0–346)	273 (1–21214)	287 (17–3667)	22 (0–528)	10 (0–659)
Honduras	43 (2–584)	14 (0–301)	225 (1–18551)	218 (12–2962)	17 (0–427)	8 (0–573)
French Guiana	17 (1–221)	5 (0–115)	90 (0–7035)	108 (6–1389)	8 (0–200)	3 (0–221)
Trinidad and Tobago	14 (1–180)	4 (0–94)	72 (0–5729)	64 (4–830)	5 (0–120)	3 (0–176)
Suriname	10 (1–126)	3 (0–65)	51 (0–3989)	44 (3–572)	3 (0–82)	2 (0–122)
Belize	9 (1–122)	3 (0–64)	49 (0–3888)	36 (2–472)	3 (0–68)	2 (0–118)
Uruguay	4 (0–51)	1 (0–27)	19 (0–1607)	14 (1–193)	1 (0–28)	1 (0–49)
Chile	3 (0–41)	1 (0–36)	17 (0–1294)	14 (1–172)	1 (0–25)	1 (0–40)
Total	12329 (713–162343)	3861 (99–84746)	64354 (170-5159256)	61231 (3541–807354)	4676 (29–116261)	2281 (8–159271)

The economic impact is measured in thousand USD. The following countries were considered in the analysis but the model estimated a risk lower than 0.01 cases per month and a negligible economic burden: Anguilla, Antigua and Barbuda, Aruba, Barbados, Bahamas, Bermuda, Bonaire, Saint Eustatius and Saba, British Virgin Islands, Cayman Islands, Clipperton Island, Curacao, Dominica, Grenada, Guadeloupe, Martinique, Montserrat, Saint Kitts and Nevis, Saint Lucia, Saint Martin, Saint Vincent and the Grenadines, Saint-Barthelemy, Sint Maarten, Turks and Caicos Islands, US Minor Outlying Islands, US Virgin Islands.

The risk of ZIKV infection has been estimated to be larger in South America than in any other part of the world [42]. Brazil will experience the largest disease and economic burden particularly in the south-east and north-east where ZIKV infections are currently the highest in the country [1, 51]. This is not surprising given that Brazil has the largest population in LATAM (≈205 million), and its climate is conducive for year-round transmission across large urban and rural areas. Other countries with large ZIKV values are Colombia, Mexico, Venezuela and Cuba where high risk of infection has been previously estimated [8, 19, 42]. Some differences were observed in the ranking of the countries most affected by ZIKV when we compared the estimated the number of cases and the number of affected pregnancies. One notable feature of these results was Cuba which was fifth in terms of overall infections in childbearing women but ninth in terms of pregnancies due to low crude birth rate [8].

**Fig 1:**
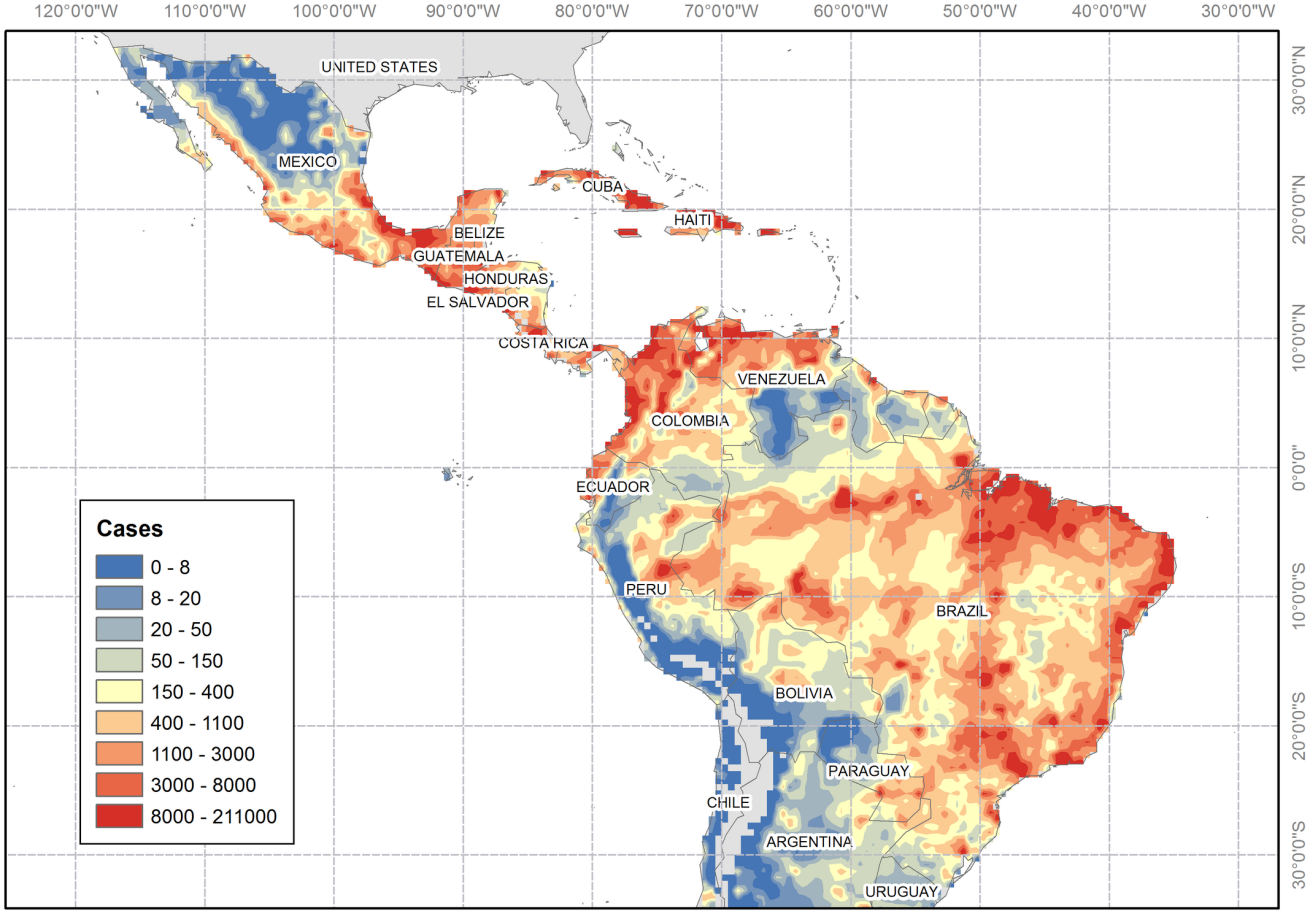
Mean monthly estimates. Estimated mean monthly Zika virus (ZIKV) cases the total population for an average year at a 0.5 × 0.5 degree resolution. Blank cells indicate risk-free areas. This Figure was created using ArcGIS Desktop 10.3 based on the model outputs projected onto a 0.5 × 0.5 grid. The shapefile for the countries was obtained using the *wrld*_*simpl* layer of the *maptools* R package.

Fig 1 shows the mean annual case predictions and indicates that the highest case estimates correspond to low elevation coastal areas of Brazil, Southern Mexico, the Caribbean, the Pacific coast of Central America, Ecuador, Colombia and Venezuela likely because the abundance of *Aedes spp*. mosquitoes declines sharply at elevations above 1,700 metres above sea level [52] due to the effect of low temperatures on the biology of the virus and the mosquito. Particularly large case numbers are expected in south-eastern and north-eastern Brazil, the Mexican Isthmus, Cuba, Puerto Rico, northern Colombia, and northern Venezuela. The estimated spatial distribution of cases agrees with observations of ZIKV infection in the region [1], with previous studies estimating the environmental suitability [10, 19, 53, 54] and risk of arboviral infection [8, 42], and with records of other arboviral diseases in LATAM [55–59]. Although not explicitly accounted for, urbanisation plays a major role in the occurrence of *Aedes*-related diseases as it increases its larval habitats [60]. Recent studies indicate that the presence of *Aedes* mosquitoes and ZIKV incidence is larger in urban than in rural areas [60, 61]. Our predicted geographical distribution of cases agrees well with such studies as the higher number of cases are predicted to occur in areas where population densities are high.

**Fig 2:**
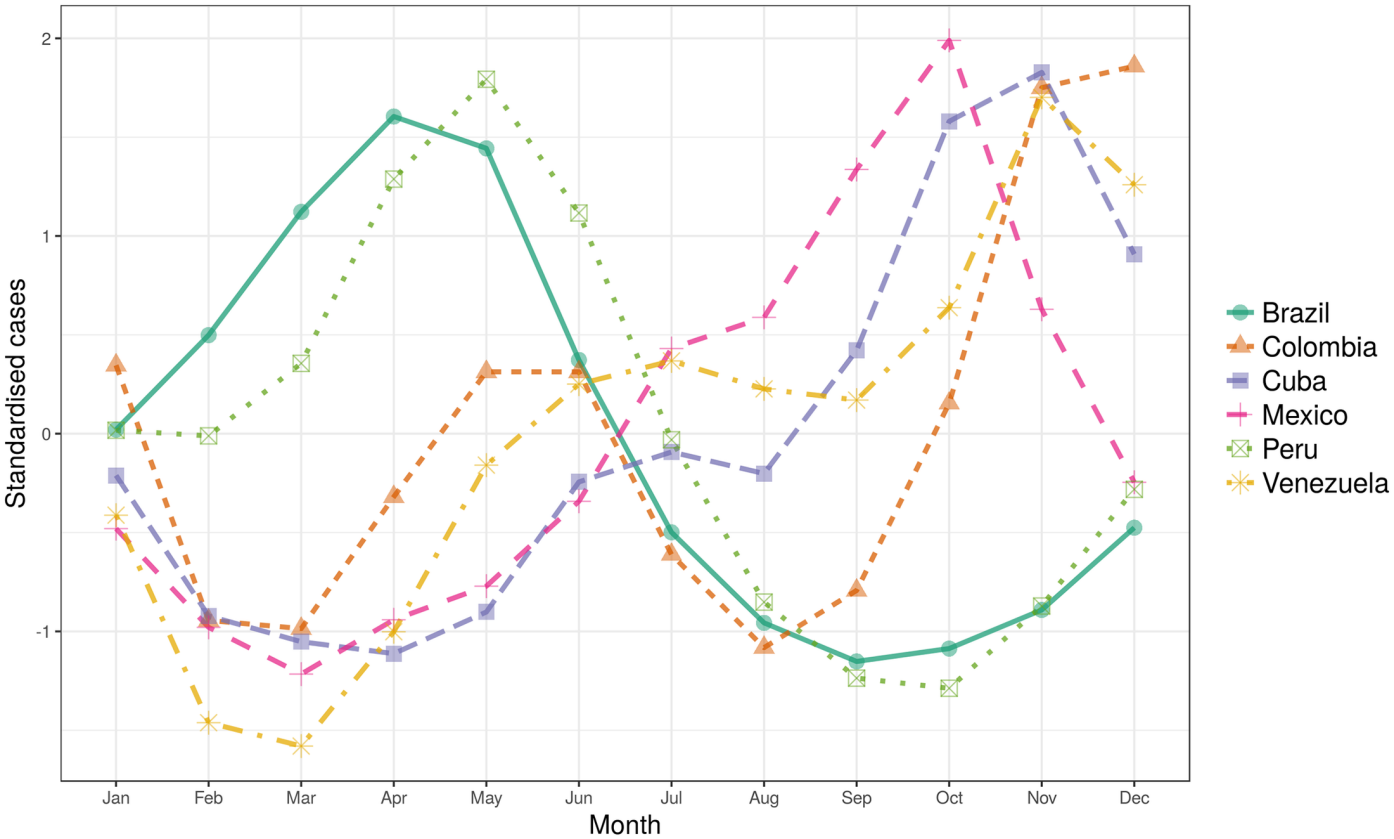
Mean annual cycle. Estimated mean annual cycle of ZIKV infections in the six countries with the highest predicted health burden.

Two distinctive seasonal cycles are observed in the predicted ZIKV cases. Fig 2 shows the estimated seasonal cycles for the six countries with the highest predicted ZIKV burden. In the southern hemisphere the high transmission season is between April and June (e.g. Brazil, and Peru), whilst in the northern hemisphere it peaks between September and November (see Mexico, Colombia, Venezuela and Cuba) in agreement with previous studies [62]. A bimodal seasonal cycle is observed in Colombia which may be related to a bimodal annual cycle of precipitation observed in the central and western regions [63]. Although precipitation was not included in the final model, by including *PET*_0:2_, we have accounted for some of its effects on ZIKV incidence. It is reminded that through evapotranspiration, atmospheric moisture returns to land as rainfall [64]. Factors such as intervention measures could also play a role in defining the seasonal trends of ZIKV transmission, yet have not been explicitly accounted for in the model.

Our modelling framework also allowed us to investigate spatio-temporal changes in ZIKV occurrence. We estimate that in the Southern hemisphere, ZIKV transmission could extend to ∼35°S between February and May, contracting thereafter to ∼30°S (see S1 Video in Supplementary material). In the Northern hemisphere, transmission remains stable up to ∼20°N most of the year, expanding to ∼30°N between July and November.

### Risk of neurological sequelae

GBS is an acute immune-mediated muscle weakness that affects the peripheral nervous system leading to paralysis that has been attributed to ZIKV infections [3, 65]. Assuming a risk of GBS between 0.24 and 31.78 cases per 1000 ZIKV infections [3, 45], and a ZIKV-dengue ratio of 0.1:1, 1:1, and 10:1, we estimate ∼64 thousand GBS cases per annum (range: 0.2–51596 thousand) across the LATAM region.

Given that the Asian lineage is related to brain developmental abnormalities [66], and that it is the lineage present in LATAM, we also estimated the potential number of microcephaly cases. Based on the crude birth rates per country [36], we estimate that ∼61 (3–807) thousand pregnancies (Table 1) could be affected by prenatal ZIKV transmission (i.e. ZIKV infection of the mother at some point during pregnancy). Assuming that only first trimester ZIKV infections may cause microcephaly, and a risk of microcephaly due to infection of between 0.88% and 14.4% [44], we estimate that ∼5 (0–116) thousand children could develop microcephaly yearly in LATAM. With an estimated direct medical cost of USD 28,818 per GBS and of USD 91,102 per microcephaly case per lifetime [46], the ZIKV-related neurological sequelae would add an economic burden of USD ∼2.3 (USD 0–159.3) billion each year. The large confidence intervals indicate that the economic impact is largely sensitive to selected zika to dengue ratio. This sensitivity has major implications for surveillance systems and public health preparedness to adequately respond to the presence of neurological sequelae.

There are uncertainties in our estimates of neurological risk. First, available data on the risk of GBS and microcephaly due to ZIKV infection are limited, especially in areas where the infection rates are unknown [44] posing problems for the use of country-specific risk factors. Second, the risk of microcephaly has dramatically increased in some locations over the past year [67] suggesting that the risk estimates should be revised relatively often. Third, the introduction of a vaccine and its combination with effective control measures could reduce the risk of infection and hence the risk of neurological sequelae.

### Epidemic-prone regions

**Fig 3:**
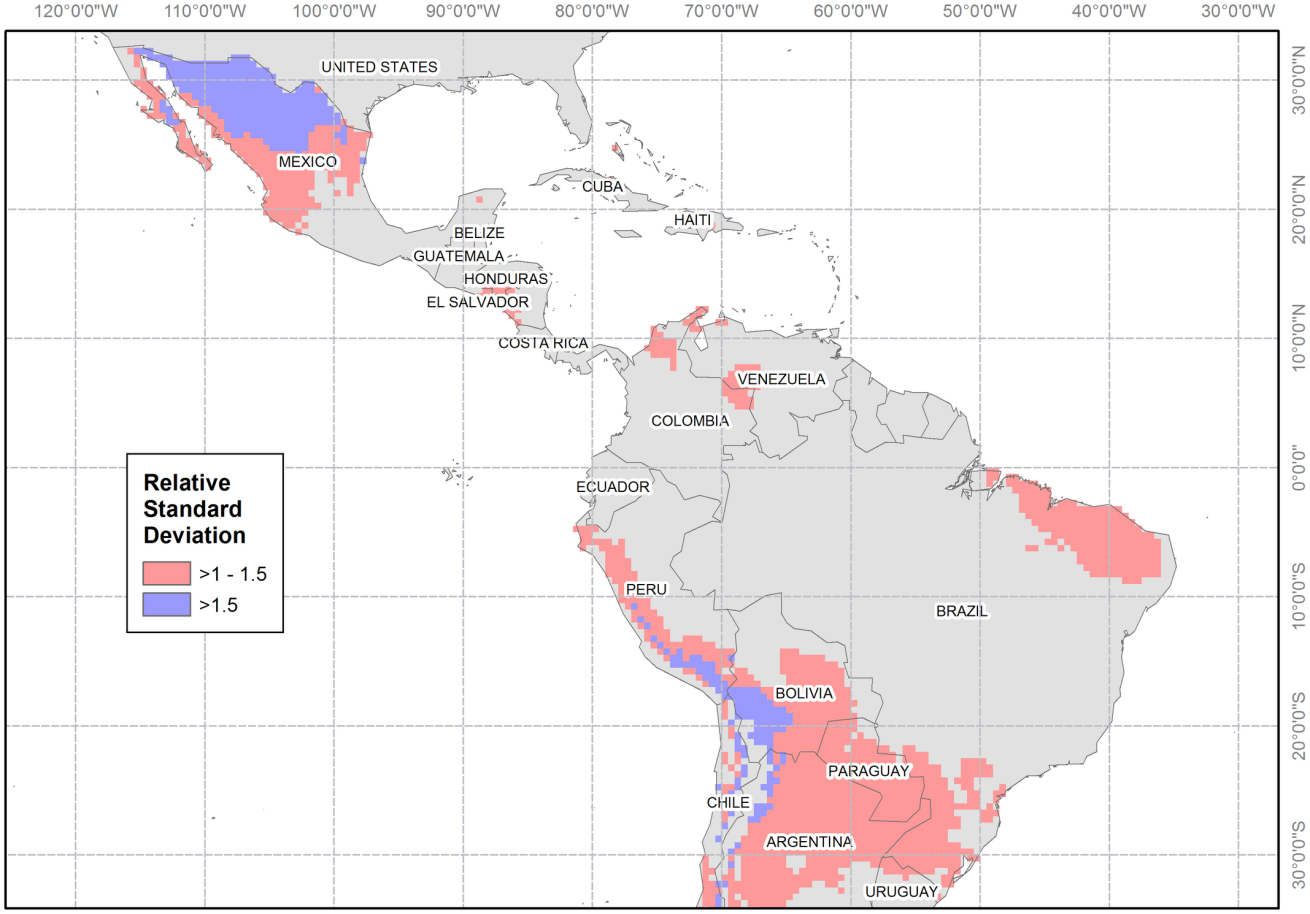
Epidemic-prone regions. Location of areas of high variability (i.e. epidemic) in Zika incidence. Blank cells indicate a relative standard deviation lower than one. This Figure was created using ArcGIS Desktop 10.3 based on the model outputs projected onto a 0.5 × 0.5 grid. The shapefile for the countries was obtained using the *wrld*_*simpl* layer of the *maptools* R package.

Under the assumption of endemicity, there are areas that will likely remain epidemic due to intermittent or short transmission seasons. Our model identified the Mexican Plateau, the Andean foothills, and parts of northern Paraguay as highly epidemic (Fig 3). Some areas with regular transmission also showed a high RSD. Cold regions (< 20°C) are marginally permissive for vector development and viral transmission [48]. Populations in these areas are likely to have low herd immunity due to low transmission intensity and viral density [68] increasing their likelihood of succumbing to epidemics. Childbearing women would therefore be more at risk in epidemic-prone areas due to low herd immunity. Areas with regular transmission may also be epidemic-prone due to outbreaks occurring earlier or later than usual with unusual high peaks in seasonal transmission as a consequence [47]. These changes may be related to variability in environmental, socioeconomic or meteorological factors [69].

### ENSO effects

**Fig 4:**
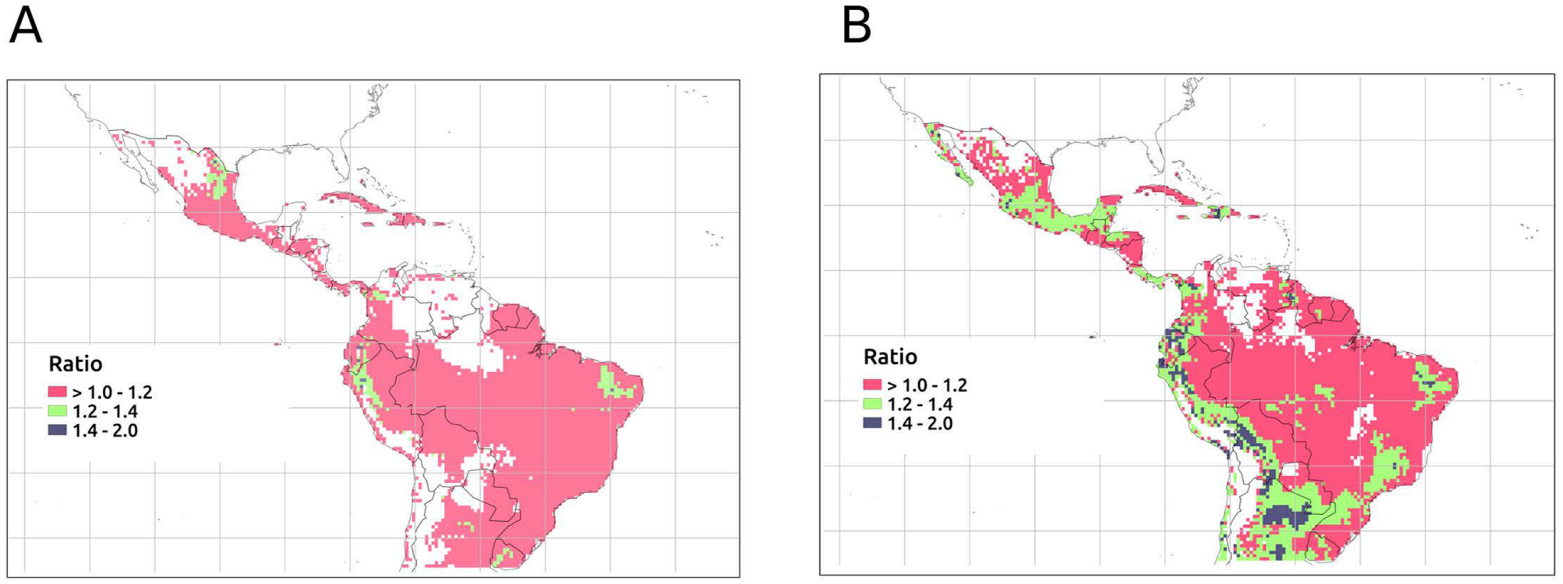
Effects of El Niño. (A) Ratio of an average El Niño month to an average non-El-Niño month. (B) Ratio of an average strong El Niño month to an average non-El-Niño month. Blank cells indicate a ratio lower than one. This Figure was created using QGIS Desktop 2.0 based on the model outputs projected onto a 0.5 × 0.5 grid. The shapefile for the countries was obtained using the *wrld*_*simpl* layer of the *maptools* R package.

A typical ENSO event is likely to increase the monthly case load across most of LATAM. Fig 4 shows the areas where increases in transmission are expected during a typical ENSO. Epidemic areas such the Andean foothills in Ecuador and Peru may show increases between 1.2 and 2.5 times the average case load of a typical non-ENSO period. During a strong ENSO event (e.g. 1997–1998 or 2015–2016), many more regions are expected to experience large increases (1.2–2.5 times the average case load during a non-ENSO period) in ZIKV cases including south-eastern Mexico, Honduras, Nicaragua and the western lowlands of South America. Previous studies indicate that ENSO could increase the risk of ZIKV infection due to an amplification effect by providing conducive conditions for transmission [42] particularly during strong events, and so ENSO may be an important driver of inter-annual variation in ZIKV transmission [70].

### Limitations

Our spatially explicit projections of ZIKV risk for LATAM provide useful information for public health preparedness. However, there are several caveats that ought to be mentioned. First, our estimates are based on the occurrence of a different organism (i.e. dengue virus) which, despite its remarkable similarities with ZIKV, has important differences [21] that may affect our results. One key difference is that there are four dengue serotypes while there is only one ZIKV serotype (subdivided into two lineages and three genotypes) [71]. None of the dengue serotypes confers protective neutralizing antibody responses against all four serotypes [6]. Thus, a single person may succumb to dengue more than once in a lifetime. ZIKV, however, induces a humoural antibody response that seems to confer lifelong immunity against reinfection, although this assumption still needs to be confirmed [72]. The assumption of a lifelong immunity to ZIKV indicates that once individuals (succumbing to the disease only once in a lifetime) become immune they also become unavailable for future infections. This situation means that recurring outbreaks would necessarily be related to the remaining susceptible individuals in a population, in addition to newly born hosts. Another important difference between the two viruses is the presence of a sexual transmission mode in ZIKV [73]. Sexual transmission could occur from asymptomatic or symptomatic individuals through genital, oral, or anal intercourse; and from male-male, male-female, and female-male contact [72, 73]. Not only sexual transmission does not occur in dengue, but also it is not driven by temperature and PET which are the main transmission drivers in our disease model. The extent to which sexual transmission can modify disease occurrence across time and space is unclear and requires further investigation.

Second, recent research has shown that the ecological niches of dengue and ZIKV are significantly different, with the niche of ZIKV expanding more than that of dengue [19]. Therefore, the potential distribution of ZIKV could expand a greater geographical area than that predicted by our model [19]. Third, our results do not account for the potential deployment of a vaccine which would significantly reduce the risk of ZIKV infection. Recent studies suggest that two ZIKV vaccine prototypes recently entered a phase-1 human-safety testing [74] and an epitope-focused vaccine for viruses in the so-called ZIKV-dengue super serogroup could be developed soon [22]. However, large-scale efficacy trials and the mass production of such a vaccine may still be years away [75].

Fourth, in the absence of long-term datasets for compareable viruses, we have based our estimates of ZIKV on one of the largest and more spatially diverse dengue datasets (accounting for over 60% of the reported cases across LATAM); however, local socioeconomic determinants of disease (e.g. access to protective measures, intervention deployment, urbanisation indices, and international travel data) in countries not included in our dataset may significantly alter disease occurrence [16]. This issue is important because socioeconomic factors vary at fine scales for political or administrative reasons and so our model could over or under estimate the risk of infection in some regions. This fact highlights the need for spatially explicit, high-resolution, publicly available epidemiological and socioeconomic time series data for LATAM.

There are remarkable genomic and epidemiological similarities between dengue virus and ZIKV [21, 22]. Based on such similarities, we have used a detailed panel of time series of contrywide dengue reports as a surrogate for ZIKV infection to estimate the potential health and economic burden across LATAM under the assumption of endemicity. The geographic distribution of other vector-borne diseases sharing the same vector [53, 54], the lack of a vaccine [5], the absence of effective vector control measures [11], and the environmental suitability of the region [10] suggest that ZIKV will likely become endemic throughout LATAM in the near future. This hypothesis concurs with a recent study that on the basis of a numerical model predicts that the virus will eventually become endemic [9]. Recent declarations from the WHO also suggest that ZIKV infection will become endemic [76]. We produced to our knowledge, the first high-resolution spatially-explicit projections of future ZIKV cases under the assumption of endemicity. Across LATAM, our projections suggest that ZIKV may impose a health burden of ∼12 (1–162) million cases per year, ∼69 (0–5276) thousand of which are likely to have major neurological sequelae. The economic burden imposed across the LATAM region amounts to USD ∼2 (0–159) billion per year, and this may increase up to ∼2 times in the aftermath of a strong ENSO event particularly in epidemic areas where public health systems are unprepared for major outbreaks. These projections can inform public health preparedness and response, and offer an opportunity to enhance capabilities in LATAM.

## Supporting information

S1 Fig Model estimates. GAMM-estimated monthly ZIKV cases for the period January 2001 to December 2012 for Brazil and Mexico. The shaded area indicates the 95% confidence intervals.
(TIF)

S2 Fig Smoothed relations. GAMM-estimated relationships between average monthly incidence and (A) temperature lagged zero to two months, and (B) potential evapotranspiration (PET) lagged zero to two months. The solid lines represent the functional form of the relationship between the incidence rate and the predictor. The shaded area indicate the estimated 95% confidence intervals. The “X” axis represents variations on each predictor. The “Y” axis is labelled *s*(*cov*, *edf*) where *cov* is the name of the predictor, and *edf* are the estimated degrees of freedom of the smoother.
(TIF)

S1 Video Estimated Zika cases per month. Spatially explicit estimates of mean Zika infections per month across Latin America and the Caribbean under the assumption of an endemic state. The Figures displayed on this video were created using ArcGIS Desktop 10.3 based on the model outputs projected onto a 0.5 × 0.5 grid. The shapefile for the countries was obtained using the *wrld*_*simpl* layer of the *maptools* R package. The video was created using *ImageMagick* and converted to mp4 using *Convertio* online.
(MP4)
