# Supplementary figures and images for: After the epidemic: Zika virus projections for Latin America and the Caribbean
Source: PLoS Negl Trop Dis. 2017 Nov 1;11(11):e0006007. doi: 10.1371/journal.pntd.0006007 (PMC5683651; doi:10.1371/journal.pntd.0006007)

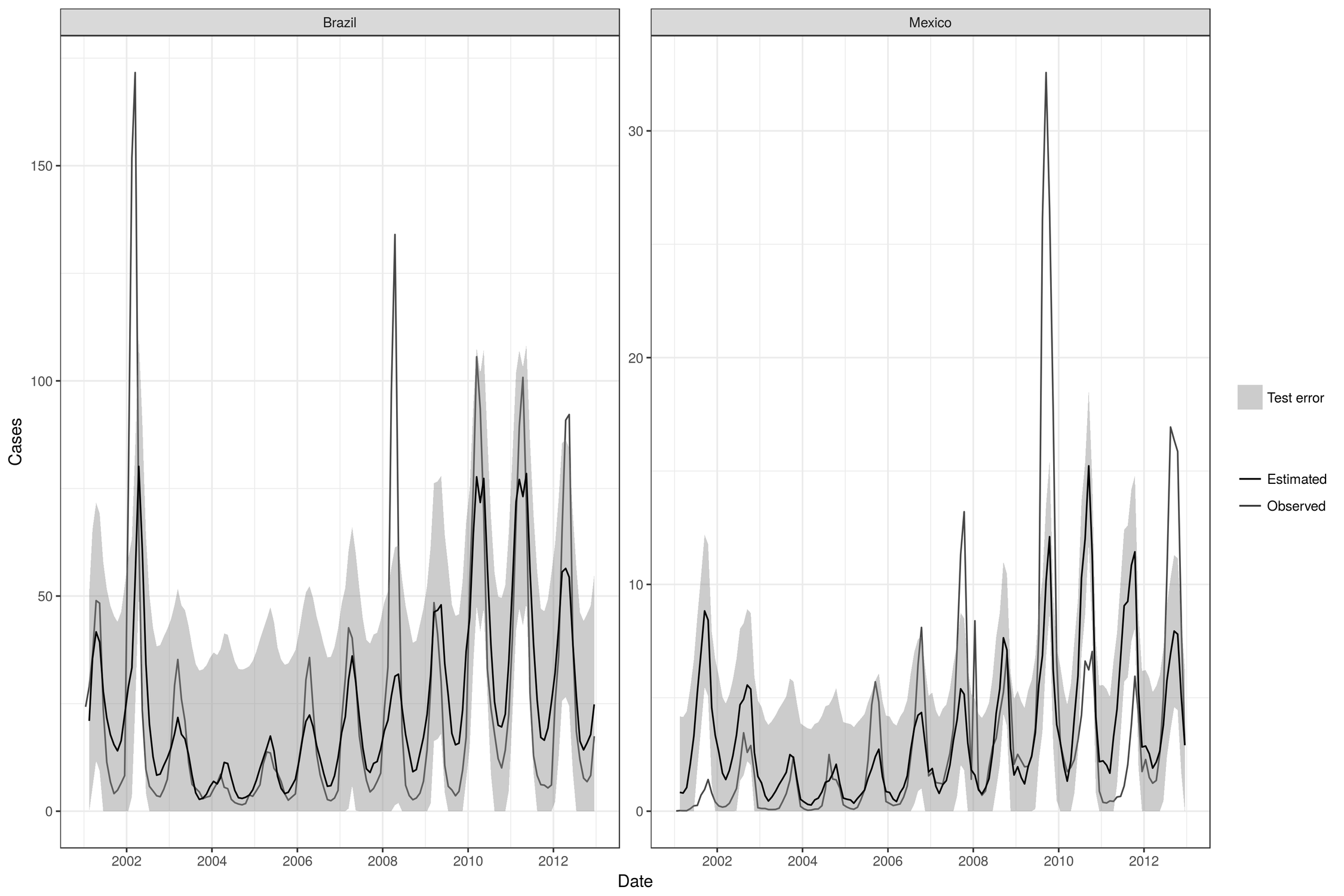

Supplement: S1 Fig — GAMM-estimated monthly ZIKV cases for the period January 2001 to December 2012 for Brazil and Mexico. The shaded area indicates the 95% confidence intervals. (TIF) [file pntd.0006007.s001.tif]

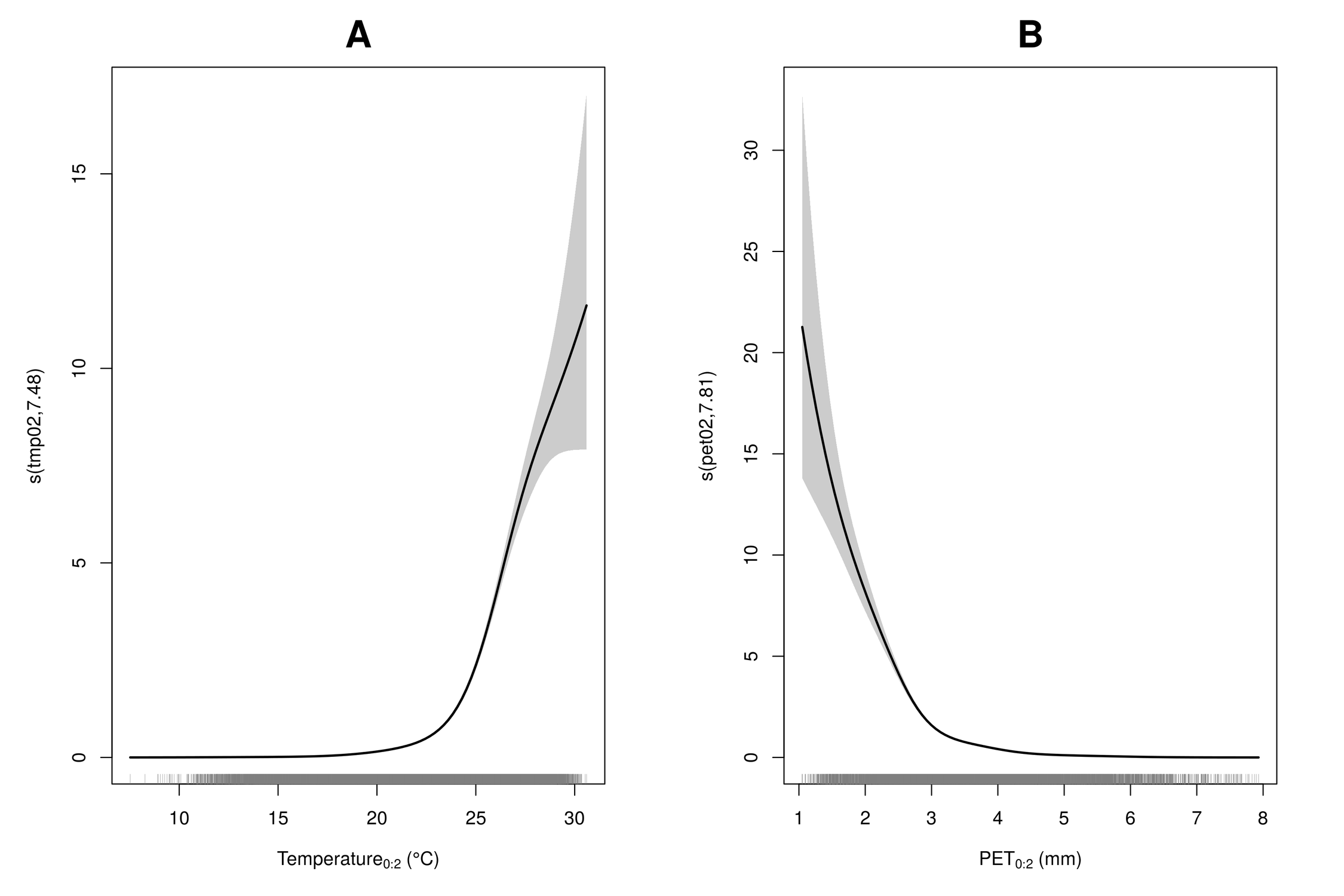

Supplement: S2 Fig — GAMM-estimated relationships between average monthly incidence and (A) temperature lagged zero to two months, and (B) potential evapotranspiration (PET) lagged zero to two months. The solid lines represent the functional form of the relationship between the incidence rate and the predictor. The shaded area indicate the estimated 95% confidence intervals. The “X” axis represents variations on each predictor. The “Y” axis is labelled s(cov, edf) where cov is the name of the predictor, and edf are the estimated degrees of freedom of the smoother. (TIF) [file pntd.0006007.s002.tif]
